# Massage as a Mode of Treatment

**Published:** 1886-12

**Authors:** 


MASSAGE AS A MODE OF TREATMENT. *By* WILLIAM MURRELL, M.D., F.R.C.P. *12mo, pp. vi, 78. Philadelphia:* P. BLAKISTON, SON & COMPANY. *Chicago:* W. T. KEENER.

This little volume contains many practical hints on the use of massage, and a perusal of it is well calculated to remove many of the too prevalent errors of the profession, regarding this subject.

It contains an excellent description of the various movements and manipulations employed in using massage, together with suggestions as to the proper choice and necessary qualifications of operators. The portion dealing with the physiological action of massage is defective, and the discussion of the chief therapeutical indications might have been lengthened with advantage.

The work as a whole has considerable value, and those not familiar with the subject can readily obtain from its pages sufficient knowledge to enable them to prescribe this mode of treatment intelligently.

At the close of the work there is an abstract of an article on "Massage and Morals," giving a brief description of some of the methods of the advertising rubber, and reiterating the statement that under no circumstances should a lady or child be treated by any one but a reliable *masseuse.* Those unacquainted with the character of many of the "professors" and "institutes" with which our larger cities are infested cannot be too heedful of this warning. H. N. M.

